# Evidence for Tonic Control by the GABA_A_ Receptor of Extracellular D-Serine Concentrations in the Medial Prefrontal Cortex of Rodents

**DOI:** 10.3389/fnmol.2017.00240

**Published:** 2017-08-02

**Authors:** Asami Umino, Sayuri Ishiwata, Hisayuki Iwama, Toru Nishikawa

**Affiliations:** ^1^Department of Psychiatry and Behavioral Sciences, Tokyo Medical and Dental University Tokyo, Japan; ^2^Center for Brain Integration Research, Tokyo Medical and Dental University Tokyo, Japan

**Keywords:** N-methyl-D-aspartate glutamate receptor, GABA_A_ receptor, glia, *in vivo* microdialysis, medial prefrontal cortex, neuron, D-serine

## Abstract

Endogenous D-serine is a putative dominant co-agonist for the N-methyl-D-aspartate glutamate receptor (NMDAR) in the mammalian forebrain. Although the NMDAR regulates the higher order brain functions by interacting with various neurotransmitter systems, the possible interactions between D-serine and an extra-glutamatergic system largely remain elusive. For the first time, we show in the rat and mouse using an *in vivo* microdialysis technique that the extracellular D-serine concentrations are under tonic increasing control by a major inhibitory transmitter, GABA, via the GABA_A_ (GABA_A_R) in the medial prefrontal cortex (mPFC). Thus, an intra-mPFC infusion of a selective GABA_A_R antagonist, bicuculline (BIC), caused a concentration-dependent and reversible decrease in the extracellular levels of D-serine in the rat mPFC without affecting those of another intrinsic NMDAR coagonist, glycine and an NMDAR agonist, L-glutamate. The decreasing effects of BIC were eliminated by co-infusion of a selective GABA_A_ agonist, muscimol (MUS) and were mimicked by a GABA_A_ antagonist, gabazine (GBZ). In contrast, selective blockade of the GABA_B_ or homomeric ρGABA_A_ (formerly GABA_C_) receptor by saclofen or (1,2,5,6-tetrahydropyridin-4-yl)-methylphosphinic acid (TPMPA), respectively, failed to downregulate the prefrontal extracellular D-serine levels. Moreover, the local BIC application attenuated the ability of NMDA given to the mPFC to increase the cortical extracellular concentrations of taurine, indicating the hypofunction of the NMDAR. Finally, in the mouse mPFC, the reduction of the extracellular D-serine levels by a local injection of BIC into the prefrontal portion was replicated, and was precluded by inhibition of the neuronal or glial activity by co-local injection with tetrodotoxin (TTX) or fluorocitrate (Fluo), respectively. These findings suggest that the GABA_A_R-mediated regulation of the D-serine signaling may exert fine-tuning of the NMDAR function and require both neuronal and glial activities in the mammalian mPFC.

## Introduction

A body of evidence has been accumulated indicating that D-serine in mammalian brains is an intrinsic coagonist for the N-methyl-D-aspartate type glutamate receptor (NMDAR) that plays a pivotal role in the expression and control of higher order brain functions (for a review see Nishikawa, 2011). The significance of D-serine is also designated by its neuroanatomical features such as the presence at high concentrations with a brain-predominant and NMDAR-like distribution throughout life and the enrichment in the cerebral cortical regions during the adult period (Hashimoto et al., 1993, 1995; Kumashiro et al., 1995). Importantly, D-serine is shown to be essential for the NMDAR activation based on observations that the selective degradation of D-serine by D-amino acid oxidase or D-serine deaminase without affecting the levels of another NMDA receptor coagonist, glycine, elicits a marked attenuation of the NMDAR channel-mediated inward current, calcium influx and cGMP formation in the forebrain areas in *in vitro* experiments (Matsui et al., 1995; Mothet et al., 2000). Disturbances in the D-serine signaling at the NMDAR has been considered to be involved in the pathophysiology of brain disorders because the D-serine depleted mice by genetic destruction of serine racemase, a D-serine synthesizing enzyme, display the NMDAR hypofunction (Benneyworth et al., 2012; Ishiwata et al., 2015) and various patterns of abnormal behavior and neuroanatomical changes including models of schizophrenia (Basu et al., 2009; Labrie et al., 2009).

The coagonist property of D-serine appears to need a specific regulatory system of the extracellular concentrations of D-serine to maintain the appropriate NMDAR activity. Although the D-serine metabolism-related molecules, such as serine racemase, D-amino acid oxidase, some neutral amino acid transporters and ionotropic glutamate receptors (Nishikawa, 2011; Ishiwata et al., 2013a,b, 2015) are found to modify the extracellular D-serine concentrations in discrete brain areas, our knowledge about the exact cellular and molecular mechanisms of control of the extracellular D-serine signaling is still limited. Especially, no studies have reported the control of the extracellular D-serine signaling by GABA neurotransmission that is a major inhibitory system in the nervous system whereas there are numerous articles on the D-serine modulation by glutamatergic transmission (Nishikawa, 2011; Ishiwata et al., 2013a). In terms of the significance of the GABA system and its balance with the excitatory glutamate system in the brain (Nestler et al., 2009), to explore the possible GABAergic control and its molecular basis, we investigated in the rat and mouse the influence of pharmacological manipulation of the various GABA receptor (GABAR) subtypes on the extracellular D-serine levels by using their respective selective antagonists and agonists in the medial prefrontal cortex (mPFC). The mPFC was chosen according to the accumulating data that this region is one of the brain areas with the highest tissue and extracellular concentration of D-serine and enriched by glutamate and GABA synapses (Nestler et al., 2009; Nishikawa, 2011).

To further clarify the role of the potential GABAergic modifications of the D-serine levels in control of the NMDAR function, we evaluated the responses of the extracellular taurine release to activation of the NMDAR (Del Arco and Mora, 1999; Oja and Saransaari, 2000; Scheller et al., 2000; Gobert et al., 2011). Because glycine and L-glutamate also augment the NMDAR function, we compared the effects of the GABAergic agents on these NMDAR-binding amino acids (Nishikawa, 2011).

Finally, to gain an insight into the cellular mechanisms of the probable GABAergic control of D-serine, we analyzed the influences of the local inhibition of the neuronal or glial activities on the interaction in the mPFC. These analyses were conducted on the basis of our previous observations pointing out that both the neurons and glia participate in the modulation of the extracellular D-serine concentrations, i.e., (1) depolarization stimuli generated by veratrine caused a prominent decrease in the extracellular contents of D-serine (Hashimoto et al., 1995); and (2) the glial activity inhibition by a reversible gliotoxin, fluorocitrate (Fluo), reduced those of D-serine (Kanematsu et al., 2006).

To achieve these objectives, we employed an *in vivo* microdialysis technique, which can locally infuse various chemicals into any brain portion, in freely moving animals combined with a concurrent quantitative assay of the chiral and non-chiral amino acids in the dialysates by high-performance liquid chromatography (HPLC) with fluorometric detection (Hashimoto et al., 1995; Ishiwata et al., 2013a,b, 2015), since monitoring the dynamics of the extracellular D-serine seems to demand the conditions preserving the interrelationships among various neurons and glia under no anesthesia (Nishikawa, 2011; Fossat et al., 2012; Rosenberg et al., 2013).

## Materials and Methods

### Animals

The present animal experiments were performed in strict accordance with the guidance of the Tokyo Medical and Dental University and were approved by the Animal Investigation Committee of the institute and the university. Male Wistar rats (Clea Japan, Japan) weighing 200–250 g or male C57BL/6J mice (Clea Japan, Inc., Japan) weighing 20–25 g between postnatal days 50 and 56 were used. The animals were housed at 23.0 ± 0.5°C in a humidity-controlled room under a 12-h light-dark cycle (lights on at 8 a.m.) and were allowed food and water *ad libitum*.

### Chemicals

(-)-Bicuculline methioide (BIC), muscimol (MUS), NMDA, *cis*-4-[phosphomethyl]-piperidine-2-carboxylic acid (CGS 19755: CGS), isoguvacine hydrochloride (IGU), gabazine (SR 95531) hydrobromide (GBZ), (*R*)-bacrofen (BAC), sacrofen (SAC), (1,2,5,6-tetrahydropyridin-4-yl)methylphosphinic acid (TPMPA) and tetrodotoxin citrate (TTX) were purchased from Tocris Bioscience (USA), and DL-fluorocitrate barium salt from Sigma-Aldrich. All other chemicals were of ultrapure grade and commercially available. The doses always refer to the free bases.

The concentration ranges from 10 μM to 250 μM of the locally applied BIC into the mPFC via the dialysis probe were selected based on the following reasons: (1) Bicuculline is usually used at the concentrations ranging from 1 μM to 100 μM in *in vitro* experiments such as bath application for the brain slices; (2) from molecular weight (MW) dependence of dialysis tube permeability and the permeability rate of D-serine or (S)-α-amino-3-hydroxy-5-methyl-4-isoxazolepropioinic acid ((S)-AMPA) with a MW 105 or 186 across the dialysis membrane, which has been estimated as approximately 14 or 10%, respectively, by HPLC analysis in our lab (at the flow rate of 2 μl/min used in this study: Hashimoto et al., 1995; Ishiwata et al., 2013b), the rate of BIC with a higher MW of 367 could be expected to be less than 10% although we could not directly detect BIC in our HPLC system; and (3) the points of (1) and (2) suggest that, in the present study, 10–250 μM of BIC in the tube could provide less than 1–25 μM outside the dialysis probe, which is within the generally used levels.

### *In Vivo* Microdialysis

The *in vivo* microdialysis was achieved using rats or mice in most or some experiments, respectively, as previously reported (Hashimoto et al., 1995; Ishiwata et al., 2013a,b, 2015). The use of two species was aimed to not only compare the possible GABAergic control of D-serine between them but also to expand the investigations on the interaction in the genetically-modified mice in the future. Rats or mice were anesthetized with pentobarbital (40 mg/kg, intraperitoneally) and mounted on a stereotaxic frame. A straight-shaped cellulose dialysis tubing (3.0 mm (rat) or 2.0 mm (mouse) in length, 0.16 mm internal diameter, MW cutoff 50,000, EICOM Co., Ltd., Japan) was then implanted into the mPFC (rat, A +3.2 mm, V +5.2 mm, L −0.7 mm; mouse, A +1.5 mm, V +3.0 mm, L −0.35 mm) according to the atlas of Paxinos and Watson (2005) for the rat or Paxinos and Franklin (2004) for the mouse. Two days after surgery, the dialysis probe was perfused with a Ringer solution (NaCl, 147 mM; KCl, 4 mM; CaCl_2_, 1.3 mM; pH 7.4) at the flow rate of 2 μl/min. After stabilizing for at least 80 min, the dialysate samples were collected every 20 min. The first three samples were used to determine the basal concentration of each amino acid. The collected samples were stored at −80°C until derivatization following the addition of D-homocysteic acid as the internal standard. After termination of the experiments, the location of the dialysis probe was macroscopically verified in each case from 150-μm-thick serial coronal slices.

The present *in vivo* microdialysis technique and its experimental conditions are able to detect the neural and glial modifications of the extracellular concentrations of the neurotransmitters, neuromodulators and their metabolite in the mPFC. Thus, our previous studies, in agreement with the results reported by other research groups (Korf and Venema, 1985; Westerink and Tuinte, 1986; Paulsen et al., 1987), ascertained that the aforementioned experimental conditions enable us to observe the following: (1) a terodotoxin-sensitive depolarization-provoked increase in the extracellular liberation of the classical amino acid neurotransmitters, L-glutamate and glycine (Hashimoto et al., 1995); (2) a remarkable diminution or complete elimination of the extracellular release of a classical neurotransmitter, dopamine, by cessation of the nerve impulse traffic or calcium chelation (Nishijima et al., 1996); and (3) alterations in the glial functioning by monitoring the extracellular L-glutamine levels (Kanematsu et al., 2006).

### High-Performance Liquid Chromatography (HPLC) Analysis

The simultaneous determination of the free amino acid enantiomers and non-chiral amino acids in the dialysate was accomplished by our previously described method using HPLC and fluorometric detection (Hashimoto et al., 1995; Ishiwata et al., 2013a,b, 2015). Briefly, the dialysate sample was derivatized with N-tert-butyloxycarbonyl-L-cysteine (Boc-L-Cys) and o-phthaldialdehyde for 2 min at room temperature. The amino acid derivative was immediately applied to the HPLC system and separated on a 4-μm Nova-Pak C18 column (300 × 3.9 mm, I.D., Waters, Japan). The column was operated at the constant flow-rate of 0.8 ml/min at 30°C. Mobile phase A was 0.1 M acetate buffer (pH 6.0) containing 7% acetonitrile and 3% tetrahydrofuran, and mobile phase B was the acetate buffer containing 22% acetonitrile and 3% tetrahydrofuran. The separation of the amino acid derivatives was performed using a linear gradient from mobile phase A to B in 53 min. The fluorescent amino acid derivatives were detected using a Waters 2475 Multi l fluorescence detector spectrofluorometer (Waters Co., Ltd.). The excitation and emission wavelengths were 344 and 443 nm, respectively.

The basal levels of the cortical dialysate D-serine, L-serine, glycine, L-glutamate, L-glutamine and taurine determined in the respective experimental groups in the present study (Table 1) are similar to each other and in good agreement with those of our previous reports (Hashimoto et al., 1995; Ishiwata et al., 2013a,b, 2015).

**Table 1:** The uncorrected absolute values of the basal extracellular concentrations of D-serine, L-serine, glycine, L-glutamate, L-glutamine and taurine in the medial frontal cortex of the rat or mouse.

Experimental groups and figure panels representing the respective data	Extracellular concentrations of various amino acids (μM)					
	D-Serine	L-Serine	Glycine	L-Glutamate	L-Glutamine	Taurine
Figures 1, 5A,B	0.97 ± 0.02 (43)	3.75 ± 0.14 (43)	5.32 ± 0.25 (43)	1.44 ± 0.14 (43)	29.40 ± 1.58 (43)	2.34 ± 0.08 (43)
[BIC, MUS]						
Figures 2, 5C,D	0.79 ± 0.02 (32)	3.69 ± 0.17 (32)	5.02 ± 0.27 (32)	2.36 ± 0.28 (32)	24.77 ± 1.37 (32)	2.22 ± 0.10 (32)
[Various GABAergic agents]						
Figure 3	1.05 ± 0.02 (31)		5.37 ± 0.45 (31)	1.48 ± 0.24 (31)		2.68 ± 0.23 (31)
[NMDA, CGS19755]						
Figures 4, 5E,F	0.80 ± 0.03 (38)	3.90 ± 0.13 (45)	4.61 ± 0.23 (45)	1.00 ± 0.11 (45)	19.61 ± 0.66 (45)	2.18 ± 0.14 (45)
[BIC, NMDA, D-serine]						
Figures 6A,B	0.48 ± 0.01 (25)				11.71 ± 043 (25)
[TTX, BIC]						
Figures 6C–F	0.48 ± 0.01 (23)				13.11 ± 0.45 (23)
[Fluo,BIC]						

*Basal amino acid concentrations show the average concentrations of each amino acid in the perfusates from the medial prefrontal cortex (mPFC) of all rats in each experimental group during the 60-min period prior to the drug treatment (three measurements were performed every 20 min). The concentrations are not corrected for recovery from the microdialysis probe. The results represent the means with SEM of the data obtained from 23 to 45 animals. The number of animals is shown in the round brackets. The chemicals used for the respective experiments are indicated in the square brackets*.

### Data Analysis

**Figure 1:**
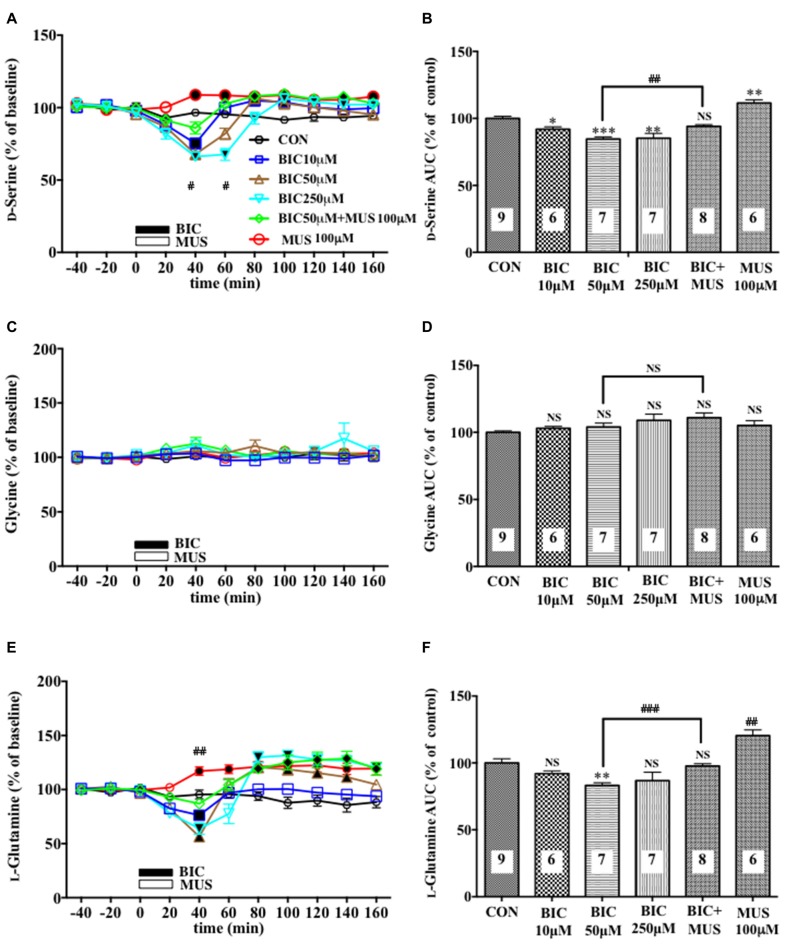
Effects of local infusion of a GABA_A_R antagonist or agonist or in combination on the extracellular concentrations of various amino acids in the rat medial prefrontal cortex (mPFC). In **(A,C,E)**, each point represents the mean with the SEM of data obtained from six to nine animals and expressed as a percentage of the basal extracellular levels of D-serine **(A)**, glycine **(C)** and L-glutamine **(E)**. The filled symbols indicate the statistically significant differences in the time point data at *P* < 0.05, 0.01 or 0.001 as compared to the Ringer solution alone-infused controls: open symbols, not significant **(A,C,E)** (D-serine **(A)**: bicuculline (BIC) 10 μM, a significant decrease (↓) at 40 min, a significant increase (↑) at 80 and 100 min; bicuculline (BIC) 50 μM, (↓) at 40 min, (↑) at 80 min; BIC 250 μM; (↓) at 40 and 60 min, (↑) at 100 and 140 min; muscimol (MUS) 100 μM, (↑) at 40–160 min; L-glutamine **(E)**: BIC 10 μM, (↓) at 40 min; BIC 50 μM, (↓) at 20 and 40 min, (↑) at 80–140 min; BIC 250 μM, (↓) at 40 min, (↑) at 80–160 min; MUS100 μM, (↑) at 40–160 min). ^#^*p* < 0.05 or ^##^*p* < 0.01 between the BIC (50 μM)-infused and the BIC (50 μM) + MUS (100 μM)-infused animals **(A,E)**. An open or filled bar followed by a drug name indicates the continuous local infusion of the Ringer solution of each drug. The area under the curve (AUC) data were calculated by cumulating each amino acid concentration for every 20-min consecutive observation from 0 min to 60 min of treatment **(B,D,F)**. *p < 0.05, **p < 0.01 or ***p < 0.001 as compared to the Ringer solution alone-infused controls. ^##^*p* < 0.01 or ^###^*p* < 0.001 between the BIC (50 μM)-infused and the BIC (50 μM) + MUS (100 μM)-infused animals. NS, not significant. The number of animals in each group is shown at the bottom of each column.

**Figure 2:**
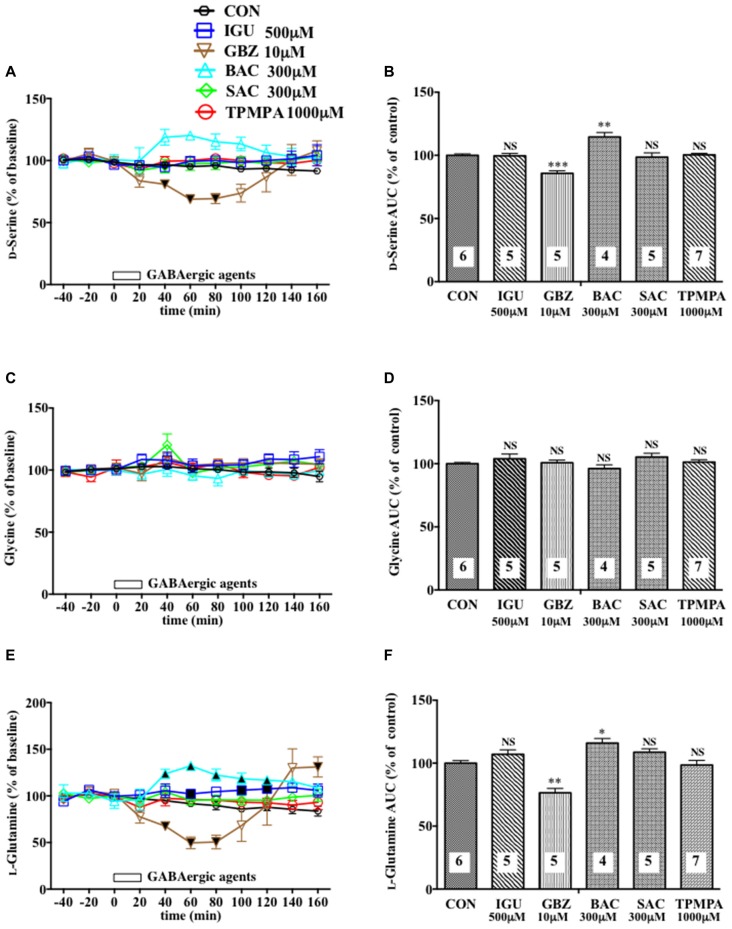
Effects of local infusion of diverse GABAergic agents on the extracellular concentrations of various amino acids in the rat mPFC. In **(A,C,E)**, each point represents the mean with the SEM of data obtained from four to seven animals and expressed as a percentage of the basal D-serine **(A)**, glycine **(C)** and L-glutamine **(E)**. The filled and open symbols indicate statistical comparisons in the same manner as described in Figure 1 (D-serine **(A)**: gabazine (GBZ) 10 μM, a significant decrease (↓) at 40–80 min; L-glutamine **(E)** isoguvacine hydrochloride (IGU) 500 μM, a significant increase (↑) at 60, 100 and 120 min; GBZ 10 μM, (↓) at 40–80 min; bacrofen (BAC) 300 μM, (↑) at 40–120 min). An open bar followed by a drug name indicates the continuous local infusion of Ringer solution of each drug. The AUC data **(B,D,F)**: *p < 0.05, **p < 0.01 or ***p < 0.001 as compared to the Ringer solution alone-infused controls. NS, not significant. The number of animals in each group is shown at the bottom of each column.

**Figure 3:**
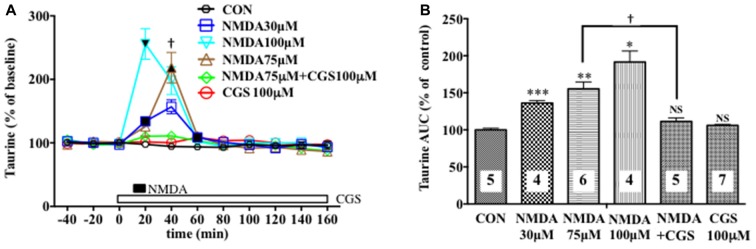
Effects of local infusion of N-methyl-D-aspartate glutamate (NMDA), CGS 19755, or in combination on the extracellular concentrations of various amino acids in the rat mPFC. In **(A)**, each point represents the mean with the SEM of data obtained from four to seven animals and expressed as a percentage of the basal taurine. The filled and open symbols indicate statistical comparisons in the same manner as described in Figure 1 (taurine: NMDA 30 μM, a significant increase (↑) at 20 and 60 min; NMDA 75 μM, (↑) at 40 min; NMDA 100 μM, (↑) at 20 min). ^†^*p* < 0.05 between the NMDA- and CGS 19755 (CGS)-infused animals **(A)**. An open, shaded or filled bar followed by a drug name indicates the continuous local infusion of Ringer solution of each drug. The AUC data **(B)**: *p < 0.05, **p < 0.01 or ***p < 0.001 as compared to the Ringer solution alone-infused controls. ^†^*p* < 0.05 between the NMDA- and CGS 19755 (CGS)-infused animals. NS, not significant. The number of animals in each group is shown at the bottom of each column.

**Figure 4:**
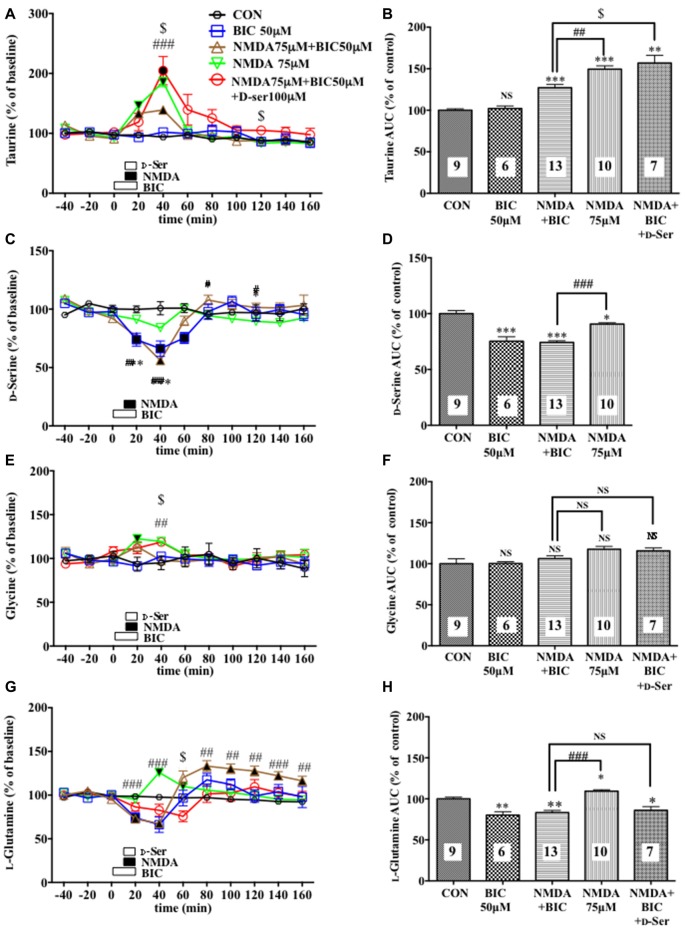
Effects of local infusion of BIC, NMDA, BIC + NMDA, or BIC + NMDA + D-serine on the extracellular concentrations of various amino acids in the rat mPFC. In **(A,C,E,G)**, each point represents the mean with the SEM of data obtained from 7 to 13 animals and expressed as a percentage of the basal taurine **(A)**, D-serine **(C)**, glycine **(E)** and L-glutamine **(G)**. The filled and open symbols indicate statistical comparisons in the same manner as described in Figure 1 (taurine **(A)**: NMDA 75 μM + BIC 50 μM, a significant increase (↑) at 20 and 40 min; NMDA 75 μM, (↑) at 20 and 40 min; NMDA 75 μM + BIC 50 μM + D-Ser, (↑) at 20 and 40 min; D-serine **(C)**: BIC 50 μM, a significant decrease (↓) at 20–60 min; NMDA 75 μM + BIC 50 μM, (↓) at 20 and 40 min; glycine **(E)**: NMDA 75 μM, (↑)at 20 min; L-glutamine: BIC 50 μM, (↓) at 20 min; NMDA 75 μM + BIC 50 μM, (↓) at 20 and 40 min, (↑) at 80–160 min; NMDA 75 μM, (↑) at 40 and 60 min). ^#^*p* < 0.05, ^##^*p* < 0.01 or ^###^*p* < 0.001 between the NMDA + BIC- and NMDA-infused animals. ^$^*p* < 0.05 between the NMDA + BIC- and NMDA + BIC + D-serine (D-Ser)-infused animals. An open, shaded or filled bar followed by a drug name indicates the continuous local infusion of Ringer solution of each drug. The AUC data **(B,D,F,H)**: *p < 0.05, **p < 0.01 or ***p < 0.001 as compared to the Ringer solution alone-infused controls. ^#^*p* < 0.05, ^##^*p* < 0.01 or ^###^*p* < 0.001 between the NMDA + BIC- and NMDA-infused animals. ^$^*p* < 0.05 between the NMDA + BIC- and NMDA + BIC + D-Ser-infused animals. NS, not significant. The number of animals in each group is shown at the bottom of each column.

**Figure 5:**
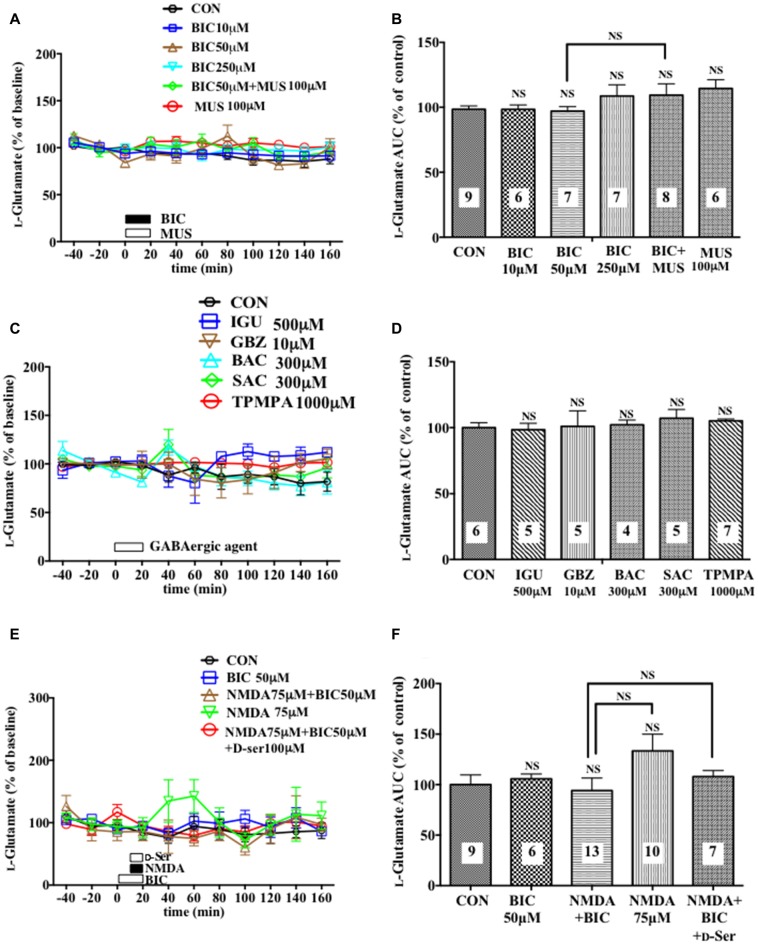
Effects of local infusion of various GABAergic agents, NMDA, D-seine or in combination on the extracellular concentrations of L-glutamate in the rat mPFC. In **(A,C,E)**, each point represents the mean with the SEM of data obtained from 4 to 13 animals and expressed as a percentage of the basal extracellular levels of L-glutamate. The open symbols indicate no statistically significant differences in the time point data at *P* > 0.05 **(A,C,E)**. An open or filled bar followed by a drug name indicates the continuous local infusion of Ringer solution of each drug. The AUC data were calculated by cumulating each amino acid concentration for every 20-min consecutive observation from 0 min to 60 min of treatment **(B,D,F)**. NS, not significant as compared to the Ringer solution alone-infused controls or between the two groups linked by the solid line. The number of animals in each group is shown at the bottom of each column.

**Figure 6:**
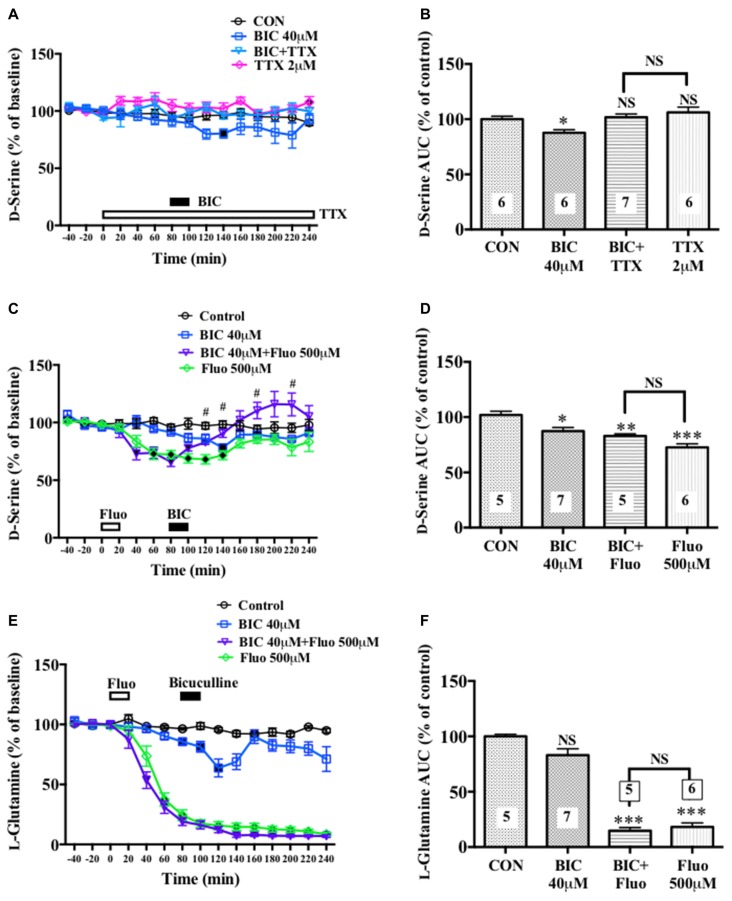
Effects of local infusion of BIC, fluorocitrate (Fluo), tetrodotoxin (TTX) or in combination on the extracellular concentrations of D-serine in the mouse mPFC. In panels **(A,C,E)**, each point represents the mean with the SEM of data obtained from five to seven animals and expressed as a percentage of the basal D-serine. The filled symbols indicate the statistically significant differences in the time point data at *P* < 0.05, 0.01 or 0.001 as compared to the Ringer solution alone-infused controls: open symbols, not significant **(A,C,E)** (D-serine **(A)**: BIC 40 μM, a significant decrease (↓) at 140 min; D-serine **(C)**: BIC 40 μM, (↓) at 140 min; BIC 40 μM + Fluo 500 μM, (↓) at 40–120 min; Fluo 500 μM, (↓) at 40–140 min; L-glutamine **(E)**: BIC 40 μM, (↓) at 80–120 min; BIC 40 μM + Fluo 500 μM, (↓) at 40–240 min; Fluo 500 μM, (↓) at 40–240 min). ^#^*p* < 0.05 between the BIC+fluorocitrate (Fluo)- and Fluo-infused animals. An open or filled bar with a drug name indicates the continuous local infusion of Ringer solution of each drug. The AUC data for 60 min after the start of the BIC infusion **(B,D,F)**: *p < 0.05, **p < 0.01 or ***p < 0.001 as compared to the Ringer solution alone-infused controls. NS, not significant. The number of animals are shown at the bottom of or above each column. It should be noted that the decreasing effects of the locally applied BIC on the extracellular concentrations of D-serine in the rat mPFC (Figures 1A,B, 4E,F) were reproduced in the mouse mPFC.

The average concentration of each substance during the period preceding the drug treatment (three measurements were performed every 20 min) was used as the baseline control value (=100%). The individual data are expressed as percentages of this baseline period. The means with SEM of the results obtained from 4 to 13 animals were calculated using the corresponding periods. The areas under the curves (AUC) of the concentration vs. time plots for the dialysate amino acids at 0–60 min of infusion from time 0 in the rat experiments (see the above “*In Vivo* Microdialysis” Section; Figures 1–5) or for 60 min after the start of the BIC (Figure 6) infusion in the mouse experiments were calculated and used as the overall measures of the treatment effects (Matthews et al., 1990).

For comparison between the two groups, statistical evaluations were performed using the unpaired two-tailed Student’s *t* test (the homogeneous variance for each experimental group) or Aspin-Welch’s *t* test (the homogeneous and heterogeneous variance for each experimental group). Statistical differences among more than three groups were estimated by Bonferroni’s method (Wallenstein et al., 1980). These statistical analyses were applied to the AUC data to clarify the exact effects of various drug treatments and their mutual differences during the specific period of each experiment (Ishiwata et al., 2013a,b). In addition, in contrast to the longitudinal view, the data of different treatment groups at each time point were similarly compared to get crosscutting view of the results of the dialysis experiments (Ishiwata et al., 2013a,b).

## Results

### Effects of Local Injection of GABA Receptor Agents on the Extracellular Concentrations of D-Serine, Glycine, L-Glutamate and L-Glutamine in the Rat Medial Prefrontal Cortex

A 20-min intra-mPFC infusion of bicuculline (BIC), a selective antagonist for the GABA_A_ receptor (GABA_A_R; Enna and McCarson, 2013), via the dialysis tubing, produced in the mPFC a significant reduction in the extracellular concentrations of D-serine in a concentration-dependent and reversible manner (Figures 1A,B) during a 0–60 min post-injection without effects on those of another NMDAR coagonist, glycine (Figures 1C,D) and an NMDAR agonist, L-glutamate (Figures 5A,B; Ogden and Traynelis, 2011). The extracellular levels of L-glutamine that have been considered to reflect the activity of glia (Hertz, 2004; Kanematsu et al., 2006) were decreased by the local BIC injection in a dose-related fashion. The reductions in the contents of the extracellular D-serine and L-glutamine were significantly inhibited by the local infusion of a selective GABA_A_R agonist, MUS (Enna and McCarson, 2013), which alone slightly increased the D-serine (Figures 1A,B) and L-glutamine (Figures 1E,F) concentrations.

Another selective GABA_A_R antagonist, gabazine (GBZ; Katzner et al., 2011; Enna and McCarson, 2013), mimicked the reducing effects of BIC on the extracellular D-serine and L-glutamine contents (Figures 2A,B,E,F) with no significant influence on the glycine (Figures 2C,D) and L-glutamate (Figures 5C,D) levels in the mPFC. However, a high effective concentration of a selective GABA_A_R agonist, isoguvacine (IGU), a GABA_B_R antagonist, sacrofen (SAC), or a selective antagonist for the ρGABA_A_R (formerly GABA_C_R), (1,2,5,6-tetrahydropyridin-4-yl)-methylphosphinic acid (TPMPA), failed to modify the frontal extracellular levels of D-serine, glycine, L-glutamine (Figures 2A–F) and L-glutamate (Figures 5C,D; Katzner et al., 2011; Ng et al., 2011; Enna and McCarson, 2013). A GABA_B_R selective agonist, bacrofen (BAC), produced a modest, but significant increase in the extracellular D-serine and L-glutamine in the cortical portion (Figures 2A,B,E,F).

### Evaluation of NMDA Receptor Function by Monitoring the Effects of the Local Injection of NMDA, an NMDA Receptor Antagonist, or in Combination on the Extracellular Concentrations of Taurine in the Rat Medial Prefrontal Cortex

We evaluated the NMDAR function under the BIC-induced reduction in the cortical extracellular D-serine contents by estimating the NMDA-evoked increase in the extracellular taurine concentrations as a well-documented index of the NMDAR responses (Del Arco and Mora, 1999; Oja and Saransaari, 2000; Scheller et al., 2000; Gobert et al., 2011). As shown in Figures 3A,B, we confirmed its reliability in that a 10-min intra-mPFC application of NMDA induced a concentration-related and reversible increase in the frontal extracellular taurine levels in a fashion sensitive to the selective NMDAR antagonist, *cis*-4-[phosphomethyl]-piperidine-2- carboxylic acid (CGS19755 (CGS), Ogden and Traynelis, 2011) given via the dialysis probe. This consequence is the first demonstration of the exact relationship between the NMDAR function and extracellular taurine levels in the local brain portion, the mPFC, which enabled us to validate the influence of the modifications of the extracellular D-serine levels on the NMDAR function by simultaneously monitoring the time course of changes in the extracellular contents of D-serine and in the NMDA-elicited upregulation of extracellular taurine in the prefrontal portion.

### Effects of a Selective GABA_A_ Receptor Antagonist on the NMDA-Induced Elevation of the Extracellular Concentrations of Taurine in the Rat Medial Prefrontal Cortex

A 20-min local infusion of BIC into the mPFC again reduced the extracellular concentrations of D-serine (Figures 4C,D), but not glycine (Figures 4E,F) and L-glutamate (Figures 5E,F), and significantly attenuated the ability of NMDA to augment the cortical extracellular taurine contents without affecting their basal levels (Figures 4A,B). This attenuation indicated hypofunction of the NMDAR by the diminished levels of the extracellular D-serine and was reversed by the intra-cortical co-infusion of D-serine (Figures 4A,B). The D-serine co-infusion did not cancel the BIC-induction of the diminution in the extracellular concentrations of L-glutamine (Figures 4G,H).

### Effects of Attenuation of Glial or Neuronal Activity on Bicuculline-Induced Decrease in the Extracellular D-Serine Concentrations in the Mouse Medial Prefrontal Cortex

A significant increase or decrease in the extracellular D-serine concentrations in the mouse mPFC was observed after neuronal activity inhibition by TTX (Figures 6A,B) or glial activity attenuation by Fluo (Figures 6C,D), respectively. The similar effects of TTX (Hashimoto et al., 1995) and Fluo (Kanematsu et al., 2006) on the extracellular D-serine levels were found in the rat mPFC in our previous studies. In addition, a locally applied BIC-induced decline in the extracellular D-serine levels seen in the rat mPFC (Figures 1A,B, 4A,B) was reproduced in the mouse (Figures 6A–D). These results suggest that rats and mice share the same control mechanisms of the extracellular D-serine signaling.

We further found that an intra-mPFC infusion of TTX (Figures 6A,B) completely abolished the diminishing effects of BIC on the frontal extracellular D-serine contents. When Fluo was locally administered for 20 min in advance (Figures 6C,D), the local BIC infusion did not downregulate, but tended to upregulate, the AUC of the D-serine levels for 60 min from the start of the BIC infusion (time points of 80–140 min) in the mPFC (Figure 6D). Under the condition of pretreatment with Fluo, there was a significant increase in the dialysate D-serine contents in the BIC + Fluo-perfused animals as compared to the Fluo alone-applied animals at time points of 120, 140, 180 and 220 min (Figure 6C). The results from the Fluo infusion experiments indicate that turning down the glial activity by Fluo not only eliminates the ability of BIC to reduce the extracellular D-serine contents but also confer the elevating effects on the contents to BIC.

## Discussion

By using an *in vivo* dialysis technique, we have for the first time provided evidence for the GABAergic control of D-serine signaling by revealing that an intra-mPFC infusion of a selective GABA_A_R antagonist, BIC, causes a concentration-dependent, reversible and a GABA_A_ agonist-sensitive decrease in the extracellular concentrations of D-serine, but not glycine or L-glutamate, in the cortical area of rats and mice. The decreasing effects are mimicked by another GABA_A_R antagonist, GBZ, but not by the GABA_B_R or ρGABA_A_R agents. The reduction in the extracellular D-serine contents following the BIC infusion leads to diminution of an NMDA-evoked increase in the extracellular taurine concentrations, indicating hypofunction of the NMDAR. The GABA_A_R agonist, MUS or GBZ, produces a slight increase or no change in the extracellular D-serine levels, respectively. These data suggest that the GABA_A_R may be implicated in a tonic and weaker phasic accelerating regulation of the extracellular release of D-serine in the mPFC.

The differential influences of the GABA_A_R antagonists on the D-serine, glycine and L-glutamate contents and the GABA_A_R antagonist-selective decreasing effects on the concentrations of D-serine in the frontal extracellular fluid exclude the possibility that the GABAergic control of D-serine seen in our experiments might be a nonspecific phenomenon. GABA_A_R antagonists, such as picrotoxin, BIC and gabazine at high doses have been reported to act on the strychnine-sensitive inhibitory glycine receptors in the retina and artificially expressed in HEK cells, depending on the composition of their subunits (Wang and Slaughter, 2005). However, the results that the reducing effects of BIC on the extracellular D-serine contents are reversed by a selective GABA_A_R agonist, MUS, that lacks the substantial effects on the inhibitory glycine receptor (Lee et al., 2011; Cinelli et al., 2016) strongly support the GABA_A_R-mediated nature of the effects of BIC. This view is also consonant with the data obtained from the mammalian cerebral cortex that BIC has been shown to inhibit the strychnine-insensitive GABA-induced CI- flux, but failed to affect the strychnine-sensitive glycine-induced CI- flux (Engblom et al., 1996).

The fact that the diminished extracellular levels of D-serine by the GABA_A_R blockade without changes in those of another NMDAR coagonist, glycine and of an NMDAR agonist, L-glutamate, elicited the NMDAR hypofunction supports the concept that D-serine may play a key role in the activation of the NMDAR as a major intrinsic coagonist for the glutamate receptor in the rodent forebrain (Nishikawa, 2011; Ogden and Traynelis, 2011). These data also deny the assumption that the NMDAR hypofunction might chiefly be caused by reduced stimulation of the glutamate site of the NMDAR. This link between the GABAergic control of D-serine and NMDAR function found in the mPFC of freely moving animals extrapolates the view that the GABAergic regulation of D-serine signaling could constitute an essential system for the physiological fine-tuning of the NMDAR activity.

It has been suggested that D-serine and glycine differentially act at the synaptic and extrasynaptic NMDARs, respectively (Papouin et al., 2012). Because the attenuated NMDAR function resulted from the selective reduction in the D-serine, but not glycine, levels after BIC infusion in the mPFC, and because NMDA activates synaptic and extrasynaptic NMDARs, the extracellular D-serine controlled by GABAergic transmission could centrally act on the synaptic NMDARs.

The exact molecular and cellular mechanisms underlying the tonic facilitatory control by the GABA_A_R of the extracellular D-serine levels await further elucidation. The activation of GABA_A_Rs could elicit D-serine release from certain cortical neurons by exocytosis because: (1) GABA causes noradrenaline exocytosis from noradrenergic nerve terminals in the hippocampus (Fassio et al., 1999); and (2) some cortical neurons in the rat are shown to be immunostained with a D-serine specific antibody (Ding et al., 2011; Balu et al., 2014). Alternatively, the excitatory influence of the GABAergic axo-axonic cells that has been reported in the rat and human cortex (Szabadics et al., 2006) might be implicated in the presently identified GABAergic control of D-serine. Furthermore, the facilitatory influence can be produced by GABA_A_R-mediated attenuation of the non-GABAergic inhibitory neurons. It cannot be excepted that the blockade of the GABA_A_R would result in reduction of the D-serine release by the increasing extracellular L-glutamate contents through liberating certain glutamatergic neurons from inhibitory regulation via their GABA_A_R because, we found the downregulating effects of an AMPA glutamate receptor agonist on the prefrontal extracellular D-serine levels (Ishiwata et al., 2013b). However, this mechanism is negated by the absence of upregulation of the extracellular L-glutamate contents following local application of the GABA_A_R antagonists (Figure 5).

The decrease in the frontal extracellular D-serine levels by the GABA_A_ antagonists is more likely to be associated with the reduced activity of a group of glial cells because these GABA_A_ antagonists also produced a parallel drop in the extracellular L-glutamine concentration (Figures 1E,F, 2E,F, 4G,H) that is a marker for the glial activity (Kanematsu et al., 2006). In agreement with this presumption, we have observed that attenuation of the glial activity by a local infusion of Fluo decreases the basal levels of the extracellular D-serine and L-glutamine (Kanematsu et al., 2006; Figures 6C–F), and blocked the ability of BIC to decrease the D-serine contents (Figures 6C,D) in the mPFC. The reducing effects of the GABA_A_R antagonists on the L-glutamine levels favor the view that the glia controlling the extracellular D-serine may be under tonic facilitation by the GABA_A_R in the mPFC. Consequently, it is possible that D-serine could be released from a population of the frontal GABA_A_R-expressing glial cells. The GABA_A_Rs on astroglia, oligodendroglia and their precursor cells have indeed been shown to be excitatory in nature as stimulation of the GABA_A_Rs of these glia depolarize these cells by the efflux of Cl^−^ ions due to the status that glial cells maintain much higher concentrations of the Cl^−^ ion in the cytoplasm than in the extracellular fluid (Verkhratsky and Steinhäuser, 2000; Lin and Bergles, 2004). In this postulated cellular setup, the recovery of NMDAR functioning (Figures 4A,B) with no significant changes in the extracellular L-glutamine levels (Figures 4G,H) after the addition of D-serine in the presence of BIC appears to agree with the idea that the exogenous D-serine might restore the NMDAR response (Figures 4A,B) by direct stimulation of its glycine site without reinstating the activity of the assumed glial cells possessing the GABA_A_Rs.

The contrasting increased extracellular contents of L-glutamine after a local application of MUS (Figure 1E), BAC (Figure 2E), and NMDA (Figure 4G) may mirror activation of certain glial cells, and are consistent with the previous observations that local or systemic application of GABA (Meier et al., 2008), GABA_B_R agonists including BAC (Gobert et al., 2011; Gould et al., 2014) and L-glutamate (Rao et al., 2003) augmented glial activity and/or the extracellular L-glutamine release in *in vitro* or *in vivo* experiments. No change or a slight decrease in the extracellular D-serine concentrations subsequent to an infusion of the GABA_B_R antagonist, SAC (Figure 2A) or NMDA (Figure 4B), repectively, suggest that GABA_B_R and NMDAR could control the glial cells that are not under influence of the GABA_A_R.

The frontal GABAergic interneurons could thus impinge on the hypothetical GABA_A_R-equipped glia and be required for the putative tonic facilitatory control over these glial cells. In support of this cellular connection, cessation of the nerve impulse flow by TTX eliminates the decreasing effects of BIC on the extracellular D-serine contents (Figures 5A,B). However, unlike the BIC perfusion, the inhibition of the GABAergic transmission by TTX failed to decrease the extracellular levels of D-serine. Although the precise reason for this inconsistency is still unclear, the non-decreasing effects of TTX could be explained by the simultaneous blockade by TTX of the unknown cortical neurons that bring a sustained inhibitory influence on the D-serine-regulating glia.

**Figure 7:**
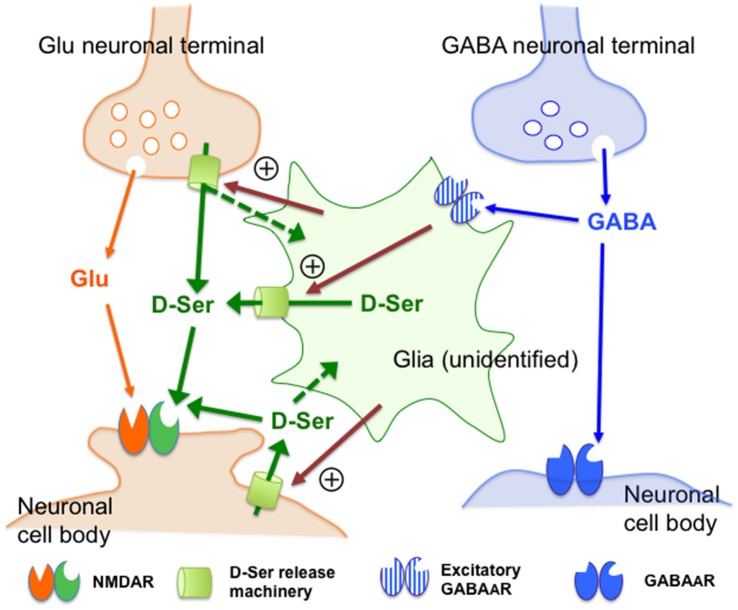
Schematic representation of the possible mechanisms of the control of the extracellular D-serine by the GABA_A_ receptor. This figure visualizes the views regarding how GABA_A_ receptors regulate D-serine release (see the explanations and arguments in the (“Discussion” Section). Together with the observations indicating that D-serine could be released from certain types of neurons or glia, the neuronal and glial activity-dependent nature of the GABA_A_R-mediated control of D-serine suggests that undefined D-serine release machineries (D-Ser release machinery: yellow-green cylinders) in the glia or neurons might be stimulated by facilitatory signals (magenta arrows) under regulation of the excitatory GABA_A_R-expressing glia that are targets of the GABA neurons in the mPFC. The dotted green arrows indicate the hypothetical transfer systems of D-serine from neurons to glia. Abbreviations: Glu, glutamate; GABA, γ-aminobutyric acid; D-Ser, D-serine. The “+” sign surrounded by circle indicates facilitatory control. The assumed excitatory GABA_A_R-expressing glia, molecular machineries and facilitatory signals for D-serine release have not yet been identified.

On the basis of the data discussed above, we schematically summarize the hypothetical cellular and molecular compositions and their interrelationships involved in the control by GABA_A_R of the extracellular D-serine in Figure 7. D-Serine could be released from certain neurons or glial cells because: (1) D-serine-like immunoreactivity has been shown in neurons (Benneyworth et al., 2012; Balu et al., 2014); (2) the cerebral white matter enriched with glial cells contains high concentrations of D-serine (Kumashiro et al., 1995); and (3) Fluo-induced inhibition of glial activity reduces the extracellular D-serine contents (Kanematsu et al., 2006). Together with these observations, the neuronal and glial activity-dependent nature of the GABA_A_R–mediated control of the extracellular D-serine levels suggests that undefined D-serine release machineries (Figure 7: yellow-green cylinders) in the glia or neurons might receive facilitatory signals (Figure 7: magenta arrows) under regulation of the excitatory GABA_A_R-expressing glia that are targets of the GABA neurons in the mPFC. Considering the differential expressions of various GABA_A_R subunits in astrocytes, oligodendrocytes and NG2 glia (Renzel et al., 2013; Balia et al., 2015; Arellano et al., 2016), and the localization of serine racemase in neurons (Balu et al., 2014), further investigations by using selective agents and specific antibodies for the respective GABA_A_R subunits, mice with cell type-specific conditional modifications of GABA- and D-serine-related genes, measures of the extracellular GABA concentrations or in combinations would clarify the accurate cell setups for the GABAergic regulation of D-serine release. Since recent studies have revealed that serine racemase, a D-serine synthese, is exclusively localized in neurons (Benneyworth et al., 2012; Balu et al., 2014), the putative glial D-serine could be supplied from neurons by some transfer systems that are indicated by the dotted green arrows in Figure 7.

From the viewpoint of the indispensable role of D-serine in the activation of the NMDAR by L-glutamate, the GABAergic control of the extracellular D-serine signal could work as an indirect regulatory equipment for the NMDAR and constitute a way to harmonize the glutamatergic excitatory and GABAergic inhibitory transmission. Based on this hypothesis, the reduced GABAergic tone can prevent the resulting over-acceleration of the NMDAR function, which causes pathological changes in the brain, e.g., neuronal cell death and degeneration (Inoue et al., 2008) and pain stimulation (Ren et al., 2006), by diminishing the extracellular D-serine concentrations at the glycine site of the NMDAR. On the other hand, the lack of effects of the GABAergic agents used in this study on the cortical extracellular concentrations of L-glutamate and glycine (Figures 1C,D, 2C,D, 4E,F, 5A–F) indicate that the initial step of the regulation of the glutamate transmission by GABARs could be the newly uncovered GABAergic control of D-serine.

It is also noted that the NMDA perfusion into the mPFC evoked a small, but significant decrease in the prefrontal extracellular D-serine levels. This phenomenon appears to be in line with the D-serine reduction subsequent to the AMPA type glutamate receptor stimulation (Ishiwata et al., 2013b) and the GABA_A_R blockade (Figures 1A,B, 2A,B, 4C,D), suggesting that these D-serine modulations through distinct receptors may assemble a process to prevent overactivation of the NMDAR.

In conclusion, the present findings demonstrate that the GABA_A_R exerts a tonic and weaker phasic facilitatory control of the extracellular D-serine levels in a neuronal and glial activity-dependent fashion in the mammalian mPFC. We propose the following mechanisms underlying the GABA_A_R-mediated regulation of D-serine: (1) GABA derived from the prefrontal cortical GABAergic neurons may continuously impinge on the excitatory GABA_A_R expressed on a group of glia that liberate D-serine; and (2) interruption of this tonic GABAergic transmission at the GABA_A_R would induce a decline in the extracellular release of D-serine by disfacilitation of these glial activities. The GABAergic control of D-serine could be composed of an unreported type of feedback system that can compensate for the deficit in the GABAergic inhibitory neurotransmission by attenuation of the excitatory input by the NMDAR. This hypothesis could explain that the GABAergic deficits may lead to hypofunction of the NMDAR in certain neuropsychiatric disorders such as schizophrenia. Therefore, advancing the research to explain the exact molecular and cellular components of the GABA-D-serine interaction should contribute to a deeper understanding of the biological basis of the neurological and mental functions and dysfunctions and to the development of novel strategies for their diagnosis and treatment.

## Author Contributions

AU and TN established the methods for the *in vivo* microdialysis technique and the semi-automatic quantitative analysis of the chiral and non-chiral amino acids by HPLC with fluorometric detection, which were used in the present study. AU, SI and HI performed the animal experiments and HPLC measurements of various amino acids, wrote their protocols and parts of the preliminary draft of the manuscript, figures and a table and undertook the statistical analysis under the guidance of TN. TN conceived, designed and directed this project, and wrote the final version of the manuscript. All authors contributed to and have approved the final manuscript.

## Conflict of Interest Statement

The authors declare that the research was conducted in the absence of any commercial or financial relationships that could be construed as a potential conflict of interest.
